# CpG Adjuvant in Allergen-Specific Immunotherapy: Finding the Sweet Spot for the Induction of Immune Tolerance

**DOI:** 10.3389/fimmu.2021.590054

**Published:** 2021-02-23

**Authors:** Guillem Montamat, Cathy Leonard, Aurélie Poli, Ludger Klimek, Markus Ollert

**Affiliations:** ^1^ Department of Infection and Immunity, Luxembourg Institute of Health, Esch-sur-Alzette, Luxembourg; ^2^ Department of Clinical Research, Faculty of Health Sciences, University of Southern Denmark, Odense, Denmark; ^3^ Centre for Rhinology and Allergology, Wiesbaden, Germany; ^4^ Department of Dermatology and Allergy Centre, Odense University Hospital, Odense, Denmark

**Keywords:** allergen-specific immunotherapy, CpG-oligonucleotides, allergy, tolerance induction, immune tolerance

## Abstract

Prevalence and incidence of IgE-mediated allergic diseases have increased over the past years in developed and developing countries. Allergen-specific immunotherapy (AIT) is currently the only curative treatment available for allergic diseases that has long-term efficacy. Although AIT has been proven successful as an immunomodulatory therapy since its beginnings, it still faces several unmet needs and challenges today. For instance, some patients can experience severe side effects, others are non-responders, and prolonged treatment schedules can lead to lack of patient adherence and therapy discontinuation. A common strategy to improve AIT relies on the use of adjuvants and immune modulators to boost its effects and improve its safety. Among the adjuvants tested for their clinical efficacy, CpG oligodeoxynucleotide (CpG-ODN) was investigated with limited success and without reaching phase III trials for clinical allergy treatment. However, recently discovered immune tolerance-promoting properties of CpG-ODN place this adjuvant again in a prominent position as an immune modulator for the treatment of allergic diseases. Indeed, it has been shown that the CpG-ODN dose and concentration are crucial in promoting immune regulation through the recruitment of pDCs. While low doses induce an inflammatory response, high doses of CpG-ODN trigger a tolerogenic response that can reverse a pre-established allergic milieu. Consistently, CpG-ODN has also been found to stimulate IL-10 producing B cells, so-called B regulatory cells (Bregs). Accordingly, CpG-ODN has shown its capacity to prevent and revert allergic reactions in several animal models showing its potential as both preventive and active treatment for IgE-mediated allergy. In this review, we describe how CpG-ODN-based therapies for allergic diseases, despite having shown limited success in the past, can still be exploited further as an adjuvant or immune modulator in the context of AIT and deserves additional attention. Here, we discuss the past and current knowledge, which highlights CpG-ODN as a potential adjuvant to be reevaluated for the enhancement of AIT when used in appropriate conditions and formulations.

## Introduction

### Allergy as a Global Health Issue

The main role of the immune system is to protect the host against external pathogens such as bacteria, viruses, fungi, or parasites. Immune cells are able to discriminate pathogens from self- and harmless external antigens, thus inducing protective immunity to pathogens and tolerance toward self- and harmless non-self-antigens (1–3). Dysregulation of immune tolerance may lead to immune-mediated diseases such as autoimmunity, cancer and various immediate-type allergic disorders (4, 5). Allergic diseases are characterized by diverse clinical disease phenotypes and symptoms, ranging from airway disorders such as allergic rhinitis and asthma (6) to systemic anaphylaxis as in the case of food and insect venom allergy (7, 8), or skin eczema/atopic dermatitis (9). Although some allergic diseases are mild and can be controlled with symptomatic medication, a considerable number of patients are at risk of life-threatening episodes (10). For instance, allergic asthma can become chronic and dramatically dampen respiratory function for life. Despite the disparity of allergic manifestations and symptoms, allergic diseases share underlying molecular and cellular mechanisms characterized by a T helper cell type 2 (Th2) response and the production of allergen-specific immunoglobulin E (IgE) by plasma cells (11). In recent decades, allergy has become a major health issue (12). Prevalence and incidence of IgE-mediated allergic diseases have been increasing over the past years in both developed and developing countries (13), thus impacting on the well-being of millions of patients worldwide and causing high socioeconomic costs (14, 15).

### Treatment of Allergic Diseases

Allergic diseases have been treated with a wide range of drugs for systemic or topical application. The main strategy used for many years has been the reduction of allergic symptoms by antagonizing the activity of main allergic mediators through inhibition of their receptors, such as the histamine type-1 (H1) receptor or the cysteinyl-leukotriene receptor type-1 (CysLTR1), by H1-antihistamines or CysLTR1 antagonists. Particularly for allergic rhinitis, nasal sprays containing antihistamines are a preferable option for patients to achieve quick and fast symptom relief (16). When allergic rhinitis becomes chronic and unresponsive to systemic or topical antihistamines, intranasal glucocorticoids are added in addition to antihistamines to target T cells and thus reduce the Th2-mediated allergic airway inflammation. Inhaled corticosteroids are also the most common therapeutic option recommended for the treatment of chronic and persistent allergic asthma. However, when the disease is refractory to this treatment, more targeted approaches are recommended such as the use of biologics. Indeed, anti-IgE monoclonal antibodies like omalizumab have shown beneficial effects in uncontrolled allergic asthma (16). Other disease-modifying biologics in asthma treatment target Th2 cytokines or their receptors (17), showing good results in the clinic (18). Although symptomatic therapies are the first line of action for patients with allergic diseases, their benefit comes with several downsides. Patients have to be treated continuously and upon treatment discontinuation, symptoms commonly relapse (19). Some of the symptomatic anti-allergic drugs have undesirable side effects such as somnolence in the case of first generation antihistamines (20). In the past years, a variety of new symptomatic medications, which show less side effects and a more targeted activity, have been developed. Among them are new generations of H1-antihistamines (21) and disease-modifying biologic monoclonal antibody therapies that interfere with the mediators and effectors of the Th2 response (17, 18, 22). However, the higher costs of such novel therapeutic options are quite often prohibitive for many patients as healthcare systems are restrictive regarding the reimbursement of advanced but more expensive treatments (23).

### Allergen-Specific Immunotherapy

AIT consists in delivering increasing doses of the allergen over time with the aim of reaching immune tolerance and clinical non-reactivity to the allergen. By activating long lasting immunomodulatory mechanisms, AIT has long-term effects such as providing sustained relief of symptoms and reducing the need for symptomatic medication (24, 25). Although AIT is a successful therapeutic strategy with dozens of licensed products worldwide, it has some unmet needs to overcome (26), since not all patients experience a significant symptom relief after therapy (27) and AIT imposes a long course with multiple doses (up to 3-5 years), which can lead to lack of patient adherence and thus treatment discontinuation (28). In addition, some patients may experience side effects during the course of AIT, ranging from mild rashes to severe anaphylaxis (29, 30). For these central reasons, AIT needs to be optimized, to make it safer, shorter in time and more successful for a maximum number of allergic patients. Another significant advantage that argues in favor of AIT is its long-term cost-effectiveness compared to symptomatic treatments.

### Mechanisms of Allergen Specific Immunotherapy

The establishment of immune tolerance by AIT implies modifications of the immune response such as induction of Tregs and B regulatory cells (Bregs), strengthened by immunological memory (31). The induction of Tregs and their essential role in the regression of the disease have been observed in humans and in mice (32). Tregs exert their immune modulatory properties through several synergistic mechanisms such as IL-10, IL-35 and TGF-β secretion (33, 34), as well as through cell surface receptors such as cytotoxic T-lymphocyte-associated protein 4 (CTLA-4), lymphocyte-activation gene 3 (LAG3), programmed cell death protein 1 (PD-1) and T cell immunoglobulin and ITIM domain (TIGIT) (35–37). Tregs might not be sufficient to reduce allergic inflammation under some circumstances (4). Indeed, Tregs have been shown to act in synergy with Bregs to promote immune tolerance. Bregs compose a specific B cell subtype that produces the anti-inflammatory cytokines IL-10 and/or IL-35 (38–41). They have been found to synthesize allergen-specific IgG4 in AIT treated patients (42, 43). Blocking antibodies that prevent specific IgE binding to the allergen are also crucial for mediating clinical non-responsiveness as it has been shown in a recent clinical trial. Blocking the major cat allergen Fel d 1 through passive immunotherapy by injecting a single dose of two monoclonal IgG4 antibodies successfully mitigated acute symptoms in cat-allergic patients (44). IgG4 is absent in mice, but IgG1, IgG2a and IgG3 have been associated with protective effects in murine AIT models (45). IgA has also been shown to be protective by neutralizing the allergen on mucosal surfaces in the context of AIT (46). The innate immune system is mainly engaged at early stages of AIT. Dendritic cells (DCs) and especially pDCs have been described as the main cellular mediators in tolerance induction during AIT by leading to the generation of Tregs (32). DCs utilize several factors to promote Treg differentiation, which include soluble factors such as IL-10, TGF-β, or IDO (47–49), as well as cellular ligands like the inducible costimulatory ligand (ICOS-L) or the programmed death ligands 1 (PD-L1) and 2 (PD-L2) (50–52).

### Improving Allergen-Specific Immunotherapy

**Figure 1:**
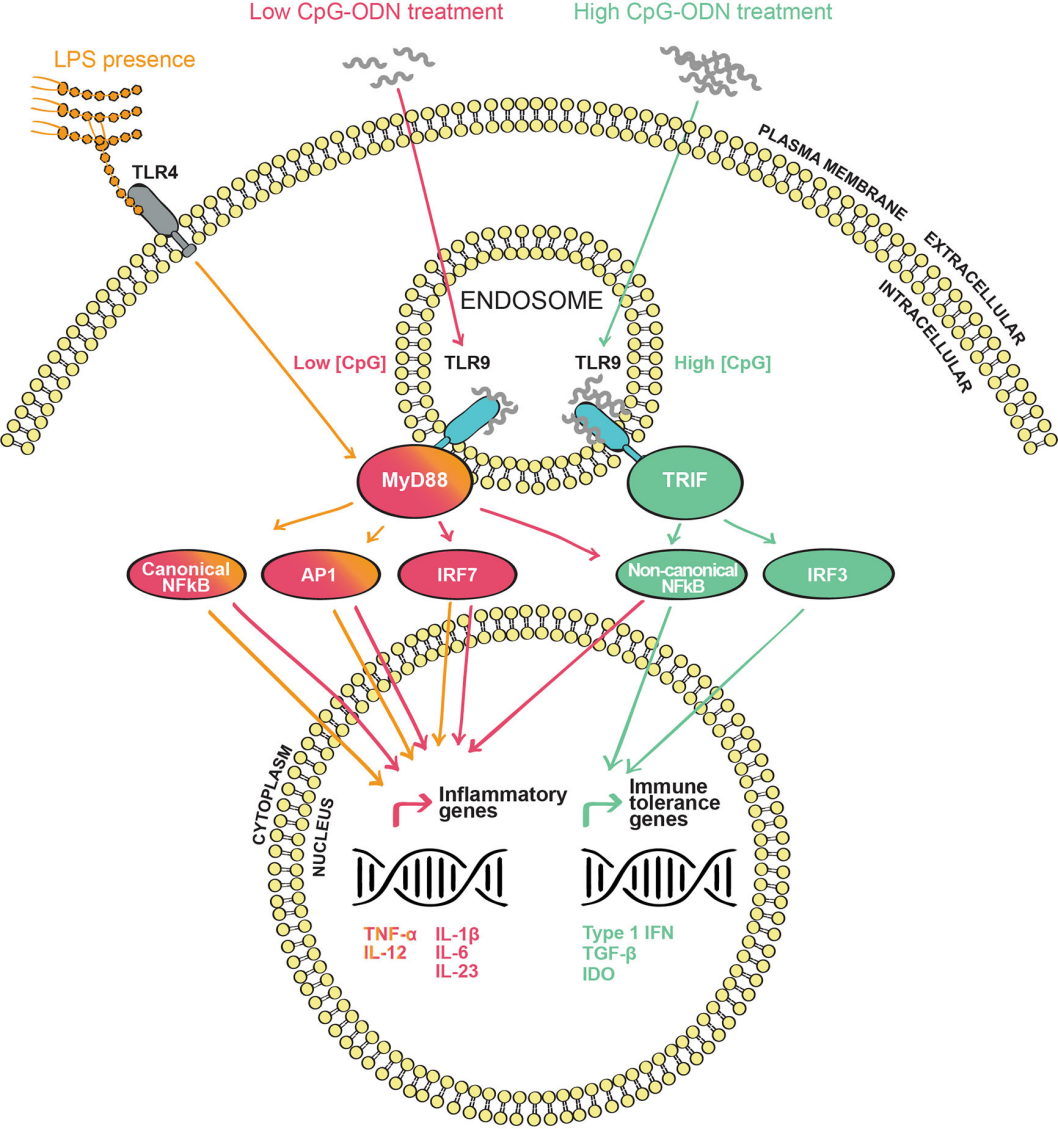
Toll-like receptor 9 (TLR9) signaling pathway(s) upon CpG oligodeoxynucleotides (CpG-ODN) stimulation: CpG-ODN is internalized through endosomes where it interacts with the endosomic domain of TLR9. In the subsequent signaling cascade, the cytosolic domain of TLR9 interacts with the adaptor proteins MyD88 or TRIF in the cytosol. Depending on the adapter engaged, a different signaling cascade occurs. MyD88 signaling (red) is triggered when TLR9 is agonized by a low CpG-ODN concentration. This cascade leads to the induction of the canonical and non-canonical NF-κB pathways, AP1 and IRF7 transcription factors, inducing inflammatory mediators such as TNF-α, IL-12, IL-1β, IL-6, and IL-23. TRIF signaling (green) is triggered when TLR9 is agonized by a high concentration of CpG-ODN. In contrast to the MyD88 signaling pathway, the TRIF cascade leads to the activation of the non-canonical NF-κB and IRF3. These transcription factors will induce the production of type 1 IFN and anti-inflammatory molecules such as TGF-β and IDO. When LPS is present (orange), TLR4 and TLR9 signals are combined to engage MyD88 leading to an enhanced inflammatory reaction by the increased production of IL-12 and TNF-α.

Several strategies have been investigated to improve AIT since its first application back in 1911 by Leonard Noon (53), among which are bypassing IgE binding, use of modified allergens or chimeric proteins, delivery of recombinant hypoallergenic proteins, new routes of administration, allergen-derived peptide immunotherapy, combination with biologics and adjuvants/immune modulators to enhance tolerance effects (4, 27). Immune adjuvanticity in the context of AIT relates to the addition of one or more compound(s) to an allergen formulation in order to improve its tolerance-inducing immunogenicity, thereby overcoming some of the unsolved needs described above. Several adjuvants have been tested over the years in the context of AIT (**Table 1**). Historically, aluminum hydroxide (Al[OH]_3_) has been the adjuvant used in many AIT formulations for its properties to form a depot (27). More recently, new adjuvants have been studied and tested for their potential to improve AIT effects and reduce its potential pitfalls. These new adjuvants are biological or synthetic compounds with a broad range of effects on the immune system such as induction of T helper cell type 1 (Th1) or regulatory T cell (Treg) responses, recruitment of dendritic cells (DCs) and other antigen presenting cells (APCs), improvement of APC uptake and signaling, or protection of the active compounds from rapid degradation (92). Adjuvants can interact with the host immune system in many ways. In the case of aluminum hydroxide, it mainly activates the inflammasome, leading to high production of the active forms of IL-1β and IL-18 (54, 55). Interestingly, aluminum hydroxide is well known to trigger a Th2 response in mice (93). Besides aluminum derivatives, toll-like receptor (TLR) ligands such as monophosphoryl lipid A (MPL), a lipopolysaccharide (LPS) derivative with TLR4 ligand properties (68), LP40, a TLR2 ligand (61), imidazoquinolines, TLR7/8 ligands (73) and CpG oligodeoxynucleotides (CpG-ODN), a TLR-9 agonist (94), have been used in AIT (**Table 1**). MPL was tested as AIT adjuvant in phase II and III clinical trials and was approved for subcutaneous immunotherapy (SCIT) of allergic rhinitis (Pollinex Quattro^®^) (95). Regarding CpG-ODN, a series of pre-clinical studies (86, 96, 97) (**Table 2**), as well as clinical trials (92, 110) (**Table 3**) were performed using this adjuvant for AIT. In 2006, data were published on a 2001 completed clinical trial using a CpG-ODN-based formulation for AIT in a randomized, double-blind, placebo-controlled phase II clinical trial for the treatment of allergic rhinitis (111). Although this novel AIT treatment appeared to have long-term clinical efficacy in patients with allergic rhinitis due to ragweed allergy (111), the formulation failed later in phase III clinical studies (26). A very low ragweed pollen exposure in the first pollen season of the phase III trial, which made it impossible to see any measurable disease in any study participant, was announced as the main reason for discontinuing the development of this novel AIT approach (122). Other possible reasons for the failure of the drug were not further discussed in the scientific literature, such as the possible presence of LPS in the purified allergen (Amb a 1), which could have interfered with TLR9 signaling (**Figure 1**), or the low concentration of CpG-ODN used. A decade later, CpG-ODN was tested as immune modulator without allergen to treat allergic rhinitis in a randomized placebo-controlled phase IIb clinical trial (113). Although the drug was proven to be successful in previous clinical trials (92, 110, 112) (**Table 3**), it did not show clinical improvement compared to placebo control (113).

**Table 1:** Comparison of adjuvants in the context of AIT.

Adjuvant name	Receptor or mechanism of action	Drug development phase	Effect on the immune system
Aluminum hydroxide (Al[OH]_3_)	Depot effect and inflammasome activation through NLRP3.	Approved for SCIT in many formulations, e.g. Alutard^®^.	**Immune tolerance induction**: No. **Inflammatory response**: Yes, general activation of immune cells and production of IL-1β and IL-18 (54, 55)^§#^. Induction of Th1 responses in PBMCs (56)^§^. **Antibody response**: yes, IgG isotypes (57)^§#^.
Tyrosine crystals	Depot effect, recognition by APC due to specific crystal size.	Approved for SCIT (MCT^®^, Acarovac Plus™) (58, 59).	**Immune tolerance induction**: Yes, increased IL-10 production by re-stimulated splenocytes (60)^#^. Higher levels of IL-10 after 1 year patient follow up (59)^§^. **Inflammatory response**: Yes, increase production of IFN-γ by both CD4^+^ and CD8^+^ antigen specific T cells (60)^#^. **Antibody response**: yes, IgG1, IgG2a, IgG2b, IgG3 isotypes (60)^#^.
LP40	TLR2	Pre-clinical	**Immune tolerance induction**: Yes, increased IL-10 production by PBMCs (61)^§^. **Inflammatory response**: Yes, increased IFN-γ, IL-6 and IL-12 production by stimulated PBMCs and splenocytes (61)^§#^. **Antibody response**: yes, IgG2a (4)^#^.
Poly I:C	TLR3	Pre-clinical	**Immune tolerance induction**: Yes, induction of IL-10 by re-stimulated splenocytes (62)^#^. **Inflammatory response**: Yes, increased production of IL-12 from DCs (63)^#^. Increased production of INF-γ by CD8^+^ antigen-specific cells (62, 63)^#^. **Antibody response**: yes, IgG1 and IgE (64)^#^.
Flagellin	TLR5	Pre-clinical	**Immune tolerance induction**: Yes, induction of IL-10 by DCs, and generation of Tregs (65)^§#^. Increased production of IL-10 by bone marrow-derived DCs (66)^#^. **Inflammatory response**: Yes, increased production of IL-6, IL-1β by bone marrow derived DCs (66)^#^. Induction of IL-4, IL-5, IL-13 and IL-17 in the lungs, as well as Th2 cell induction after airway challenge (67)^#^. **Antibody response**: yes, IgG2a (66) and IgE (67)^#^.
Monophosphoryl lipid A (MPL)	TLR4	Approved for SCIT to treat allergic rhinitis (Pollinex Quattro^®^)	**Immune tolerance induction**: No. **Inflammatory response**: Yes, increased production of IFN-γ (68)^§^. Induction of Th1 responses (69, 70)^#^. **Antibody response**: yes, IgG1 and IgG4 (71)^§^. IgG2a (70)^#^.
Imidazoquinolines	TLR7/8	Phase II (AZD8848) to treat allergic asthma (72).	**Immune tolerance induction**: Yes, increased IL-10 production by monocytes (73)^§^. Induction of tolerogenic DCs and Treg promotion (74)^#^. **Inflammatory response**: Yes, increased production of IFN-γ by allergen-specific T cells from allergic donors. Increased production of IL-12, IL-18, TNF-α, and IL-15 by monocytes derived (73)^§^. **Antibody response**: lack of IgG induction *in vitro* (75)^#^.
CpG-ODN	TLR9	Phase IIb to treat allergic rhinitis (low dose) Pre-clinical (high dose)	**Immune tolerance induction**: Yes (in high doses), increased production of IL-10 by alveolar macrophages (76)^#^, production of TGF-β and IDO by pDCs (76)^#§^, induction of Treg cells (77–81)^§#^, induction of Bregs (82)^§^ (**Table 4**). **Inflammatory response**: Yes (at low doses), increased IL-12, IFN-γ, IL-6 (83, 84)^§^. Induction of Th1 responses (85)^§^. (**Table 4**). **Antibody response**: yes, IgG1, IgG2a, IgG2c IgG3, and IgA (79, 84, 86–91)^#§^.

NLRP3, NLR family pyrin domain containing 3; BALF, bronchoalveolar lavage fluids. ^#^In mouse, ^§^In human.

**Table 2:** Summary of pre-clinical studies using CpG oligodeoxynucleotides (CpG-ODN) for allergic diseases.

Model/disease	Drug features	Drug design	CpG-ODN dose*	Administration scheme	Outcome	Reference
Mouse model of asthma to schistosome eggs.	CpG-ODN, B-class. LPS level: undetectable	CpG-ODN + allergen	1 dose: 30 µg per injection.(1.5 mg/kg)	1 IP injection 7 days before challenge (preventing treatment). 1 IP injection together with allergen (co-administration treatment).	Reduction of allergy burden in co-administration and preventive treatment. Eosinophilia, Th2 cytokines reduction and IgE reduction. IFN-γ and IL-12 increase.	Kline et al. (97)
Mouse model of asthma to rBet v 1.	CpG-ODN, B-class. LPS level: <0.1 EU/6 µg of DNA	CpG-ODN + allergen	1 dose 61.23 µg (10nmol) per injection. (3 mg/kg)	3 IP, SC or NI administrations 2 weeks before sensitization (preventive treatment). 3 IP, SC or NI administrations 2 weeks after challenge (active treatment). 3 IP injections (co-administration with allergen and Al[OH]_3_).	Reduction of allergy burden in co-administration, preventive and active treatment. Eosinophilia, Th2 cytokines, and IgE reduction. IgG2a and IgA increase.	Jahn-Schmid et al. (86)
Mouse model of asthma to ragweed extract.	CpG-ODN, B-class. LPS level: <0.02 U/kg	CpG-ODN + extract	1 dose: 35 µg per administration. (1.75 mg/kg)	1 IT administration 48h before challenge.	Reduction of allergy burden in preventive treatment. Eosinophilia, IgE and IL-4 reduction. IFN-γ, IFN-γ/IL-4 ratio and IgG2a increase IFN-γ dependent.	Sur et al. (98)
Mouse model of immune response to mosquito salivary antigen (rAed a 2).	CpG-ODN, B-class. LPS level: undetectable	CpG-ODN + allergen	10 µg, 30 µg and 90 µg per injection in the preventive treatment. (0.5 mg/kg, 1.5 mg/kg and 4.5 mg/kg respectively) 30 µg per injection in the active treatment. (1.5 mg/kg)	1 ID injection 24h before first sensitization (preventive treatment). 2 ID injections during sensitization (co-administration treatment), 1 injection every 4 weeks.	Reduction of Th2 reaction in co-administration and preventive treatment. IgE and Th2 cytokines reduction. IL-12 and IgG2a increase. Delayed-type hypersensitivity reactions in CpG-ODN treated mice.	Peng et al. (99)
Mouse model of immune response to hepatitis B major surface antigen (HBsAg)	CpG-ODN 1826, B-class. LPS level: no mention	CpG-ODN + antigen	10 µg per injection. (0.5 mg/kg)	1 IM injection 2 or 4 weeks before or after sensitization.	Reduction of Th2 reaction in preventive and active treatment. IgG1 reduction. IgG2a and CTL activity increase.	Weeratna et al. (88)
Mouse model of asthma to OVA	CpG-ODN, B-class. LPS level: undetectable	CpG-ODN + allergen	1 µg per administration. (0.05 mg/kg)	3 NI administrations: 2 weeks after sensitization, 1 administration every 2 weeks.	Reduction of allergy burden response in active treatment. Airway hyperresponsiveness, eosinophilia, IgE and Th2 cytokines reduction. IgG2 and IL-10 increase.	Jain et al. (100)
Rhesus monkey model ofexperimentally induced allergic asthma to HDM.	CpG-ODN, A-class. LPS level: ND	CpG-ODN solely	12.5 mg per administration. (1.9 mg/kg)	3 NI administrations: 24h after sensitization, 1 administration every 2 weeks.	Reduction of allergy burden in active treatment. Airway hyperresponsiveness, eosinophilia, mast cell number and tissue remodeling reduced.	Fanucchi et al. (101)
Mouse model of asthma to OVA	CpG-ODN 1826, B-class. LPS level: undetectable	CpG-ODN solely and CpG-ODN + allergen	100 µg per administration. (5 mg/kg)	3 OG administrations: 3 days before, same day of and 7 days after sensitization (preventive treatment). 6 OG administrations: 2 weeks after sensitization, 1 administration every week (active treatment).	Reduction of allergy burden in active and preventive treatment. Eosinophilia, IgG1, IgE and Th2 cytokines reduction. IgG2c increase.	Kitagaki et al. (91)
Mouse model of immune response to OVA	CpG-ODN BL07S from *Bifidobacterium longum*. B-class. LPS level: undetectable	CpG-ODN + antigen	10 µg per injection. (0.5 mg/kg)	2 SC injections: 2 weeks interval co-injected with sensitization.	Reduction of Th2 reaction in co-administration treatment. IgE and Th2 cytokines reduction. IgG2a increase.	Takahashi et al. (102)
Murine model of asthma to OVA	CpG-ODN B-class. LPS level: ND	CpG-ODN + allergen	50 µg per injection. (2.5 mg/kg)	1 SC injection: 24h after Th2 cell adaptive transfer.	Reduction of allergy burden in active treatment. Eosinophilia, mucus hyper-production, Th2 cytokines and cell migration to the lung reduction. Type I IFN dependent.	Ashino et al. (103)
Mouse model of asthma to OVA	CpG-ODN B-class. LPS level: no mention	CpG-ODN solely	100 µg per injection. (5 mg/kg)	4 IP injections: 6 days after sensitization, 1 injection every 24h.	Reduction of allergy burden in active treatment. Airway hyperresponsiveness, eosinophilia, Th2 cytokines, cell migration to the lung reduction. IgG2a and IFN-γ increased.	Chang et al. (104)
Mouse model of asthma to OVA	CpG-ODN (sensitized using custom primers). LPS level: undetectable	CpG-ODN solely	50 µg/per injection. (2.5 mg/kg)	3 SC injections: 3 days after sensitization, 1 injection every week.	Reduction of allergy burden in active treatment. Airway hyperresponsiveness, eosinophilia, IgE, IgG1 and Th2 cytokines reduction. IgG2a increase.	Fonseca et al. (105)
Mouse model of asthma to the Japanese cedar pollen allergen (Cry j 1).	CpG-ODN 1018, B-class. LPS level: <0.03 endotoxin unit/µg	CpG-ODN coupled to Cry j 1	5 µg per injection. (0.25 mg/kg)	3 SC injections: 3 days before sensitization, 1 injection every 24h.	Reduction of allergy burden in preventive treatment. IgE and Th2 cytokines reduction. IL-12, IFN-γ and IgG2a increase.	Kaburaki et al. (106)
Mouse model of asthma to *Aspergillus fumigatus* extract.	CpG-ODN 1826, B-class. LPS level: endotoxin free	CpG-ODN solely	30 µg per injection. (1.5 mg/kg)	2 IP injections: 7 days after sensitization, 1 injection every week.	Reduction of allergy burden in active treatment. IgE and Th2 cytokines reduction. IL-12, IFN-γ and IgG2a increase.	Volpi et al. (84)
Mouse model of asthma to ragweed extract	CpG-ODN 1018, B-class. LPS level: <5 endotoxin units/mg	CpG-ODN solely	20 µg per administration. (1 mg/kg)	5 to 12 NI administrations: 14 days after sensitization, 1 administration every week.	Reduction of allergy burden in active treatment. Eosinophilia, IgE and Th2 cytokines reduction. IFN-γ increase. IFN-γ dependent.	Cambell et al. (107)
Mouse model of asthma to HDM	CpG-ODN 1826, B-class. LPS level: <0.1 EU per dose	Nanoparticle-conjugated CpG-ODN	2 µg per administration. (0.1 mg/kg)	4 NI administrations: 3 days before sensitization, 1 administration every 2 days (preventive therapy). 4 NI administrations: 3 days after sensitization, 1 administration every 2 days (active therapy).	Reduction of allergy burden in preventive and active treatment. Eosinophilia, IgE, mucus production and Th2 cytokines reduction. INF-γ increase.	Ballester et al. (108)
Mouse model of asthma to cockroach extract	CpG-ODN 1826, B-class. LPS level: ND. HLPC purified	CpG-ODN solely	3 µg per administration. (0.15 mg/kg)	1 NI administration: 3 days before sensitization (preventive therapy). 1 NI administration: 3 days after sensitization (active therapy).	Reduction of allergy burden in preventive and active treatment. Airway hyperresponsiveness, eosinophilia, IgE, goblet cell hyperplasia and Th2 cytokines reduction. IL-10 and CD4^+^ Foxp3^+^ regulatory T cells increase.	Kim et al. (80)
Mouse model of food allergy to peanut extract	CpG-ODN 1826, B-class. LPS level: ND	Nanoparticle-conjugated CpG-ODN	1.8 µg per administration. (0.09 mg/kg)	4 OG administrations: 3 weeks after sensitization, 1 administration every week.	Reduction of allergy burden in active treatment. Anaphylaxis, histamine levels, IgE/IgG1 levels, and Th2 cytokines reduction. IgG2a and INF-γ increase.	Srivastava et al. (109)
Mouse model of asthma to HDM	CpG-ODN, A-class. LPS level: <24 EU/mg	CpG-ODN solely	50 µg per administration. (2.5 mg/kg)	1 NI administration: 7 days before sensitization (preventive therapy). 1 NI administration: 7 days after sensitization (active therapy).	Reduction of allergy burden in preventive and active treatment. Airway hyperresponsiveness, eosinophilia, goblet cell hyperplasia and Th2 cytokines reduction. IL-10 increase. IL-10 dependent.	Sabatel et al. (76)
Mouse model of asthma and active cutaneous anaphylaxis to OVA	CpG-ODN 2395, C-class. LPS level: endotoxin free	CpG-ODN + allergen	10 ug per injection. (0.5 mg/kg)	2 SC injections: co-administrated with the sensitization, 1 injection every week.	Reduction of allergy burden in co-administration treatment. Eosinophilia, IgE and anaphylaxis score reduction. IgG2c and IgG2a increase.	Alberca Custodio et al. (90)
Mouse model of asthma to Fel d 1	CpG-ODN 1668, B-class. LPS level: endotoxin free	CpG-ODN + allergen	21 µg per injection. (1.05 mg/kg)	3 IP injection: 14 days after sensitization, 1 injection every 2 weeks.	Reduction of allergy burden in active treatment. Airway hyperresponsiveness, eosinophilia, mast cell ratio, B cell activation and Th2 cytokines reduction. Gata3^+^ Treg increase.	Leonard et al. (79)

CTL, cytotoxic T-lymphocyte; OVA, ovalbumin; HDM, house dust mite; IP, Intraperitoneal; NI, Nasal instillation; IT, Intratracheal instillation; ID, intradermal; IM, intramuscular; OG, oral gavage; ND, not determined. *20 g mean weight for a mouse and 6.5 kg mean weight for a rhesus monkey were considered to calculate the dose.

**Table 3:** Summary of CpG oligodeoxynucleotides (CpG-ODN) clinical studies for allergy and other diseases.

Disease	Study phase	Identifier	Drug information	CpG-ODN dose*	Administration scheme	Outcome and safety	Completion date	Reference
**IgE-MEDIATED ALLERGY**								
Allergic rhinitis to ragweed-pollen	Randomized double-blind placebo-controlled phase 2	NCT00346086	CpG-ODN 1018 conjugated to Amb a 1	6 increasing doses: 0.06µg, 0.3 µg, 1.2 µg, 3 µg, 6 µg and 12 µg. (0.00004 mg/kg, 0.00008 mg/kg, 0.000016 mg/kg, 0.0002 mg/kg, 0.0004 mg/kg, 0.0008 mg/kg and 0.00016 mg/kg respectively)	6 SC injections, 1 injection every week.	Long-term clinical efficacy. Drug was well tolerated with mild local injection-site reactions.	Aug 2001	Creticos et al. (111)
Perennial allergic rhinoconjunctivitis with asthma to HDM	Single center open-label phase 1/2a	NCT00652223	CYT005-AllQbG10 (combination of HDM allergen extract with CYT003-QbG10)	§1 dose: 60 µg per injection. (0.0008 mg/kg)	6 SC injections, 1 injection every week.	Patients achieved practically complete alleviation of allergy symptoms. Drug was well tolerated with mild local injection-site reactions.	Nov 2005	Senti et al. (110)
Perennial allergic rhinoconjunctivitis to HDM	Randomized double-blind placebo-controlled phase 2	NCT00574704	CYT005-AllQbG10 (combination of HDM allergen extract with CYT003-QbG10)	Not identified	6 SC injections, no schedule found.	No results accompanying the study	Mar 2008	Not published
Perennial allergic rhinoconjunctivitis to HDM	Randomized double-blind placebo-controlled phase 2	NCT00574223	CYT005-AllQbG10 (combination of HDM allergen extract with CYT003-QbG10)	Not identified	8 SC injections no schedule found.	No results accompanying the study	Mar 2009	Not published
Perennial allergy to HDM and/or Cat	Randomized double-blind placebo-controlled phase 2	NCT00575003	CYT003-QbG10 (VLP filled with an CpG-ODN G10)	Not identified	6 SC injections no schedule found.	No results accompanying the study	Mar 2009	Not published
Perennial allergic rhinoconjunctivitis to HDM	Randomized double-blind placebo-controlled dose-finding phase 2b	NCT00800332	CYT003-QbG10 (VLP filled with an CpG-ODN G10)	§2 doses: 0.1 mg and 0.2 mg per injection. (0.00134 mg/kg and 0.0026 mg/kg respectively)	6 SC injections, 1 injection every week.	Higher dose of the drug improved disease symptoms and reduced medication use in allergic individuals. Drug was well tolerated with mild local injection-site reactions.	Nov 2010	Klimek et al. (92)
Allergic asthma requiring long-term treatment with inhaled corticosteroids	Randomized double-blind placebo-controlled phase 2	NCT00890734	CYT003-QbG10 (VLP filled with an CpG-ODN G10)	§1 dose: 0.18 mg of per injection. (0.0024 mg/kg)	7 SC injections. 3 first injections every week, and 4 next injections every 2 weeks.	Drug may contribute to asthma control during steroid reduction in patients. Drug was well tolerated with mild local injection-site reactions.	Nov 2010	Beeh et al. (112)
Uncontrolled moderate to severe allergic asthma on standard inhaled corticosteroids	Randomized double-blind placebo-controlled dose-finding phase 2b	NCT01673672	CYT003-QbG10 (VLP filled with an CpG-ODN G10)	§3 doses: 0.06 mg, 0.2 mg and 0.4 mg per injection. (0.0008 mg/kg, 0.00266 mg/kg and 0.0054 mg/kg respectively)	7 SC injections, 1 injection every 1 or 2 weeks.	Drug showed no additional benefit in patients. Drug was well tolerated with mild local injection-site reactions.	May 2014	Casale et al. (113)
**CANCER**								
Chronic lymphocytic leukemia	Randomized open label phase 1	NCT00233506	CpG-ODN 7909	2 doses: (0.15 mg/kg 1.05 mg/kg)	4 to 8 IV and SC injections, 1 injection every week.	Drug was well tolerated with mild side-effects such myalgia, malaise, and fevers.	Jun 2011	Zent et al. (114)
Refractory solid tumors	Randomized open label phase 1b	NCT03052205	CpG-ODN IMO-2125 (Tilsotolimod)	Maximum dose: 32 mg/injection. (0.43 mg/kg)	6 IT injections on weeks 1, 2, 3, 5, 8, and 11.	Drug was well tolerated and no related adverse events were observed.	Oct 2019	Babiker et al. (115)
Metastatic melanoma or recurrent or metastatic head and neck squamous cell carcinoma	Randomized open labelPhase 1b/2	NCT02521870	CpG-ODN SD-101 in combination with Pembrolizumab (anti-PD1)	4 doses: 1 mg, 2 mg, 4 mg, 8 mg per injection. (0.013 mg/kg, 0.027 mg/kg, 0.053 mg/kg and 0.11 mg/kg respectively)	11 IT injections in escalating doses. 4 first injections every week, and 7 next injections every 3 weeks.	Drug was well tolerated with injection-site reactions and transient, mild to moderate “flu like” symptoms.	Feb 2020	Ribas et al. (116)
Malignant melanoma	Non-randomized open label phase 1	NCT03084640	CMP-001b (CYT003-QbG10) in combination with Pembrolizumab (anti-PD1)	§7 escalating doses: 0.2 mg, 1.5 mg. 2 mg, 2.5 mg, 3 mg, 3.5 mg and 4 mg per injection. (0.0134 mg/kg, 0.02 mg/kg, 0.026 mg/kg, 0.034 mg/kg, 0.04 mg/kg, 0.046 mg/kg and 0.054 mg/kg respectively)	7 SC injections into accessible lesion(s), 1 injection every week.	Drug in combination with Pembrolizumab was well tolerated with mild toxicities such as fever and headache.	May 2021	Milhem et al. (117)
**INFECTIOUS DISEASES**								
*Plasmodium falciparum* (Malaria) infection	Randomized phase 1	NCT00344539	CpG-ODN 7909 in combination with AMA1-C1/Alhydrogel^®^ (experimental malaria vaccine)	1 dose: 564 µg per injection. (0.0075 mg/kg)	3 IM injections, 1 injection every 4 weeks.	Drug was well tolerated with mild reactions such as local and systemic adverse events.	Jan 2007	Mullen et al. (118)
*Bacillus anthracis* (Anthrax) Infection	Randomized Double-blind placebo-controlled phase 1	NCT01263691	CpG-ODN 7909 in combination with BioThrax (FDA-licensed anthrax vaccine)	1 dose: 0.5 mg per injection. (0.0067 mg/kg)	2 IM injections, 1 injection every 2 weeks.	Drug was well tolerated with mild injection-site reactions.	Jun 2012	Hopkins et al. (119)
Hepatitis B Virus (HBV) infection	Randomizedobserver–blindedactive–controlledphase 3	NCT02117934	HBsAg-1018 (CpG-ODN 1018 in combination with epatitis B surface antigen (HBsAg))	1 dose: 3 mg per injection. (0.04 mg/kg)	2 IM injections, 1 injection every 4 weeks.	Drug was well tolerated with non-reported side-effects.	Oct 2015	Jackson et al. (120)
*Mycobacterium tuberculosis* (Tuberculosis) infection	Randomized placebo-controlled phase 1	NCT03255278	GamTBvac: CpG-ODN 2216 in combination with Ag85a and ESAT6-CFP10 *M. tuberculosis* antigens	3 doses: 37.5 µg, 75 µg and 150 µg per injection. (0.0005 mg/kg, 0.001 mg/kg and 0.002 mg/kg respectively)	2 SC injections, 1 injection every 2 months.	Drug was well tolerated with mild injection-site reactions.	Dec 2017	Vasina et al. (121)

VPL, virus-like particles; HDM, house dust mite; SC, subcutaneous; IV, intravenous; IM, intramuscular; IT, intratumoral; ^§^CpG-ODN G10 represents 20% of the CYT003-QbG10. *75kg mean body weight was considered to calculate the dose.

Thus, despite the fact that CpG-ODN-based therapies for allergic diseases have shown early clinical promise, they were not pursued further for the treatment of allergic diseases after experiencing a lack of success in later phases of controlled clinical trials (**Table 3**). Nevertheless, experimental and clinical research on CpG-ODN continued in other biomedical areas, which led to the successful FDA approval of CpG-ODN as immune adjuvant in hepatitis B vaccination in 2018 (120) and to multiple recent clinical studies, where CpG-ODN has been used as immune modulator in cancer immunotherapy (**Table 3**). In this review, we describe and discuss the current knowledge and latest results on CpG-ODN as immune adjuvant for AIT and asthma treatment. Indeed, although other adjuvants, especially TLR ligands, have shown immune-regulatory properties (123), CpG-ODN has been predominantly described for its capacity to induce immune tolerance in comparison to other adjuvants that are also used in the context of AIT (**Table 1**). Our review of the literature, which is based on searches in PubMed “(CpG ODN OR CpG oligodeoxynucleotides) AND (allergy OR allergic disease)”, “(CpG ODN OR CpG oligodeoxynucleotides) AND (immune tolerance OR tolerance induction)” and “(CpG ODN OR CpG oligodeoxynucleotides) AND (safety)” points to CpG-ODN as an adjuvant with previously unrecognized potential for AIT that is able to effectively induce immune tolerance to allergens when used at appropriate higher concentrations, which are still safe in humans. This review delineates that, based on more recent evidence, a reevaluation of CpG-ODN as immune adjuvant for AIT seems to be warranted.

## CpG Oligodeoxynucleotides (CpG-ODN)

### CpG-ODN as a TLR9 Ligand

To distinguish pathogen structures from self-antigens the immune system has tailored specific systems detecting danger signals or pathogen associated molecules patterns (PAMPs). PAMPs comprise a variety of pathogen structures such as LPS, proteins like flagellin, glycan structures, genetic material (DNA and RNA) among others (124). Immune cells can specifically identify PAMPs through pattern recognition receptors (PRRs). Bacterial genetic material, unlike vertebrate DNA, is enriched of unmethylated deoxycytidyl-deoxyguanosine (CpG) dinucleotides (125). These PAMPs of unmethylated bacterial CG motifs are recognized by the Toll-like receptor 9 (TLR9) (126). Experimentally, short synthetic oligodeoxynucleotide (ODN) sequences containing unmethylated CpG dinucleotides (CpG-ODN), mimicking bacterial DNA signatures, are used to agonize TLR9, thereby avoiding any other TLR co-activation by bacterial contaminants (127).

### Different Classes of CpG-ODN

So far, at least four types of CpG-ODN have been identified, which induce different effects on the immune system (**Table 4**). Thus, it is crucial to know the immunomodulatory properties of each CpG-ODN type when formulating CpG-ODN-based treatments. A-class CpG-ODN has a phosphodiester/phosphorothioate backbone containing a single CpG motif which is flanked by palindromic sequences, plus a poly-G string located at the 3’ end. B-class CpG-ODN harbors multiple CG doublets within a phosphorothioate backbone (136), the latter providing resistance to nuclease digestion increasing its lifetime by 6-fold compared to the phosphodiester backbone of A-class (83). C-class was reported more recently as a new CpG-ODN. C-class is a combination of both afore-mentioned CpG-ODN classes. As B-class, it is built entirely on phosphorothioate nucleotides but it resembles the A-class by its palindromic CpG motifs (133). The last and more recent CpG-ODN group that was described is the P-class, which, unlike the other CpG-ODN types, contains two palindromic sequences that facilitate the formation of higher-ordered structures (135).

**Table 4:** CpG classes: sequences, structure, interaction with Toll-like receptor 9 (TLR9), and response induced in immune cells.

CpG-ODN type	Sequence example	Structure	Effect on Immune cells
A-class (D-type)	GGTGCPuPy **CG** PuPyGCAGGGGGG	Single CpG motif flanked by a palindromic sequence, plus a 3’ Poly-G end. Mixed phosphodiester/phosphorothioate backbone.	• IFNγ production by NK cells (83)^§^ • Lytic activity by NK cells (128)^§#^ • Type 1 IFN production by pDC (85, 129, 130)^§#^
B-class (K-type)	AT**CG**ACTCTCGAG**CG**TTCTC	Multiple CpG motifs. No palindromic sequences. Phosphorothioate only backbone.	• IgM, IL-6 production by B cells and B cell proliferation (83, 131)^§^ • Type 1 IFN production by pDC (85)^§^ • pDC maturation (130)^§^ • Th1 response induction (85)^§^ • NK cells activation (85)^§^ • Production of TGF-β and IDO by pDC (84, 132)^§#^ • Tolerance induction by pDC (77, 84, 132)^§#^
C-class	T**CG** T **CG** TT **CG** AA **CG** A **CG**TTGAT	Multiple CpG motifs and one palindromic sequence. Phosphorothioate only backbone	• IL-6 production by B cells (131, 133)^§^ • B cell activation and proliferation (134)^§^ • Type I IFN in pDC (130)^§^ • pDC maturation (130)^§^
P-class	T **CG** T **CG** A **CG** AT-**CG** G **CGCGCG** C **CG**	Multiple CpG motifs flanked and two palindromic sequences. Phosphorothioate only backbone.	• Type I IFN by PBMCs (135)^§^ • Increased plasma levels of type 1 IFN and IL-6 *in vivo* (135)^§^

Pu, purine nucleotide; Py, pyrimidine nucleotide; Bold, CpG motifs; Underlined, palindrome sequences; PBMCs, peripheral blood mononuclear cells. ^#^In mouse, ^§^In human.

### TLR9 Downstream Signaling and Its Modulation by CpG-ODN Concentration

TLR9 is located intracellularly, with the binding site for CpG-ODN pointing to the endosomal compartment. This specific orientation requires the internalization and endosomal uptake of CpG-ODN to agonize TLR9 and trigger its signaling cascade (129) (**Figure 1**). Once TLR9 is activated by CpG-ODN binding, its cytosolic domains, known as Toll/IL-1 receptor (TIR) domains, are stimulated and signaling is transmitted further. TIR domains interact with the common TLR adaptor protein myeloid differentiation factor 88 (MyD88) (137), the classical adaptor protein attributed to many TLRs. Subsequently, IL-1R-associated kinases 1, 2 and 4 (IRAKs) and tumor necrosis factor receptor-associated factor 6 (TRAF6) are phosphorylated (124). From this point, several mitogen-activated protein kinases (MAPK) and other kinases such as IκB kinases (IKK) are sequentially recruited. Those kinases facilitate the translocation to the nucleus of various transcription factors such as the nuclear factor kappa-light-chain-enhancer of activated B cells (NF-κB), the activator protein-1 (AP-1) or the interferon regulatory factor 7 (IRF7) (138–140). These transcription factors activate the transcription of pro-inflammatory genes such as *TNF, IL-1B, IL-1A* and interferon type 1 (*IFNA* and *IFNB*) genes (129, 141). Indeed, type 1 IFN production by plasmacytoid dendritic cells (pDC) in response to TLR9 activation has been demonstrated to be dependent on the MyD88-IRF7 signaling cascade (129). The kinetics of the gene expression signature induced by CpG-ODN are complex. It has been shown that only 30 min after injection of CpG-ODN *in vivo* a panel of genes is upregulated, with a peak of expression around 3 h after administration. Interestingly, a second delayed peak is observed 5 days post administration (141), demonstrating that CpG-ODN can have short-, mid- and long-term effects. This fact is not to be neglected when using CpG-ODN for therapeutic purposes. A few years ago, the TIR domain containing adaptor inducing IFN-β (TRIF) was demonstrated to signal upon TLR3 and TLR4 activation (142–144). More recently, Volpi et al (84). showed that TLR9 can also signal through TRIF besides the classic MyD88 pathway (**Figure 1**). In fact, this group demonstrated that TLR9 signals through TRIF when a high concentration of CpG-ODN is applied to pDCs, while the classical MyD88 adaptor is engaged by TLR9 when a lower concentration of CpG-ODN is used (84) (**Figure 1**). Interestingly, the divergence in TLR9 downstream signaling implies a differential gene expression signature. On the one hand, the MyD88 transduction pathway triggered both the canonical NF-κB (IKKβ-dependent) and non-canonical NF-κB (IKKα-dependent) pathways, leading to the expression of pro-inflammatory cytokines such as tumor necrosis factor alpha (TNF-α), IL-1β, IL-12, IL-6 and IL-23. On the other hand, TRIF downstream signaling activated exclusively the non-canonical NF-κB pathway and IRF3, leading to the expression of anti-inflammatory mediators such as transforming growth factor beta (TGF-β) and indoleamine 2,3-dioxygenase (IDO) (145) (**Figure 1**). This research reveals a new concept, in which TLR9 can be differentially activated depending on the ligand concentration. The distribution of TLRs on human peripheral blood mononuclear cells is variable. While TLR9 is not expressed in some human immune cells such as neutrophils and monocytes, it has a high expression in pDCs and B cells, and it is found to a lesser extent in NK cells and T cells (146). In the mouse, TLR9 exhibits similar expression and activation patterns as in humans (146–148) with subtle differences. For instance, and according with their lack of TLR9 expression, monocytes from peripheral human blood are not activated by CpG-ODN (149). However, mouse monocytes express TLR9 and respond to CpG-ODN stimulation by engaging the NF-κB pathway and the subsequent production of cytokines such as TNF-α (148, 150). With regard to the organs relevant for allergic airway disease, human and mouse alveolar macrophages express TLR9 (151). However, only human but not mouse resident lung DCs show TLR9 expression (152, 153).

Given all these indications, CpG-ODN may induce slightly different immune responses between mice and humans (151, 154). Moreover, optimal CpG-ODN sequences that agonize TLR9 differ among species (127, 131, 155). However, the effect of TLR9 activation remains essentially unchanged between species since the afore-mentioned downstream transduction elements are well conserved. Nonetheless, inter-species differences should be considered when applying CpG-ODN-based treatments in animal models, aiming at translation to humans (**Tables 4** and **5**).

**Table 5:** Sequence and type of CpG oligodeoxynucleotides (CpG-ODN) used in pre-clinical studies (**Table 2**) and clinical trials (**Table 3**).

CpG-ODN name or study	Species used	Sequence	CpG-ODN type/structure
**A-Class**			
G10 (156)	Human	5′-GGGGGGGGGGGACGATCGTCGGGGGGGGGG-3′	A-class
CpG-ODN 2216	Human	5′-GGGGGACGATCGTCGGGGGG-3′	A-class
Fanucchi et al. (101)	Mouse	5′TGACTGTGAACGTTCGAGATGA-3′	A-class
Sabatel et al., 2017 (76)	Mouse	5′-TCCATGACGTTCCTGATGCT-3′	A-class
**B-Class**			
1018 ISS (CpG-ODN 1018)	Human and mouse	5′-TGACTGTGAACGTTCGAGATGA-3′	B-class
CpG-ODN 7909 = CpG-ODN 2006	Human	5′- TCGTCGTTTTGTCGTTTTGTCGTT -3′	B-class
Kline et al. (97), Jain et al. (100)	Mouse	5′-TCCATGACGTTCCTGACGTT-3′	B-class
Jahn-Schmid et al. (86)	Mouse	5′-ATCGACTCTCGAGCGTTCTC-3′	B-class
Sur et al., 1999 (98)	Mouse	5′-GCTAGACGTTAGCGT-3′	B-class
Peng et al. (99)	Mouse	5′-TCCATGACGTTCCTGACGTT-3′	B-class
CpG-ODN 1826	Mouse	5′-TCCATGACGTTCCTGACGTT-3′	B-class
CpG-ODN BL07S	Mouse	5′-GCGTCGGTTTCGGTGCTCAC-3′	B-class
CpG-ODN 1668 (79)	Mouse	5′-TCCATGACGTTCCTGATGCT-3′	B-class
Chang et al. (104)	Mouse	5′-TCCATGACGTTCCTGACGTT-3′	B-class
**C-Class**			
IMO-2125 (Tilsotolimod)	Human	5′-TCG*AACG*TTCG*-X-G*CTTG*CAAG*CT-3′	C-class
SD-101	Human	Not described.	C-class
CpG-ODN 2395	Mouse	5′-TCGTCGTTTTCGGCGCGCGCCG-3′	C-class
**Others**			
MGN1703 (Lefitolimod)	Human	CTAGGGGTTACCACCTTCATTGGAAAACGTTCTTCGGGGCGTTCTTAGGTGGTAACCC by dimer-circularization	Double-stem loop immunomodulators (dSLIM) (157, 158)
Fonseca et al. (105)	Mouse	Not described.	Not described.

### CpG-ODN Effect on Immune Cells

CpG-ODN influences many immune cell types, especially APCs such as DCs and macrophages. Dendritic cell precursors express various TLRs, among which is TLR9 (149), thus enabling their activation by CpG-ODN (159). As pDCs express high levels of TLR9, their response to CpG-ODN has been studied in detail. CpG-ODN enhances pDC migration to lymph nodes and stimulates their production of cytokines such as IL-6, TNF-α, type 1 interferons and granulocyte-macrophage colony-stimulating factor (GM-CSF) (160). Moreover, CpG-ODN induces pDCs to express several co-stimulatory surface molecules such as CD40, CD54, CD80, CD86 (85, 161–163). Very interestingly, unmethylated CG motifs have also been shown to promote an anti-inflammatory phenotype in pDCs. CpG-ODN-treated pDCs can induce CD8^+^ Tregs that express immune inhibitory molecules such as LAG3 and CTLA4 (164). Similarly, pDCs stimulated with CpG-ODN can prime naïve T cells to differentiate into CD4^+^ CD25^+^ Tregs that produce IL-10 and TGF-β (77) (145). Langerhans cells express TLR9 and migrate upon CpG-ODN intradermal injection in mice (165). Finally, a subpopulation of splenic DCs was capable of producing a potent IDO signal upon CpG-ODN IV injection, thereby acquiring a suppressive capacity similar to Tregs (166). Interestingly, it has also been shown that pDCs stimulated with C class CpG-ODN, can directly license human B cells into plasma cell differentiation and antibody production in a T cell independent way (167). B cells are also greatly influenced by CpG-ODN. Indeed, CpG-ODN is a strong B cell mitogen, induces them to produce IL-6 and IgM (127, 168), and boosts their activation and differentiation during Ig production (169). Along the same line, B cells stimulated with CpG-ODN change their Ig profile and produce different antibodies, an effect being dependent on TRL9 and MyD88 signaling (87). NK cells can react through direct or indirect signals upon CpG-ODN stimulation. Certain studies showed that NK cells cannot respond to CpG-ODN directly, but are rather triggered indirectly through soluble factors produced by CpG-ODN-activated pDCs (146). By contrast, other studies support the fact that CpG-ODN can directly induce NK cells to produce IFN-γ and increase their activity (128, 170, 171). Since T cells express very low levels of TLR9, CpG-ODN is assumed not to stimulate these cells directly. Similarly to NK cells, T cells are rather engaged *via* signals transmitted by CpG-ODN-activated pDCs (146). However, although it seems rather evident that there is no direct effect of CpG-ODN on T cells, some research points out that CpG-ODN could stimulate T cells without any intermediate, but in a TLR9 and MyD88 independent fashion (172). This was supported by another study, showing that purified human T and NK cells produce IFN-γ upon direct CpG stimulation (173). In addition, one study has shown MyD88 to be essential for direct CpG-ODN stimulation in CD4^+^ T cells (174).

In summary, each CpG-ODN type has a wide range of effects on various immune cells, among which pDCs and B cells are the most relevant ones. With regard to the use of CpG-ODN as an adjuvant for AIT, B-class CpG-ODN stands out for its capacity to induce a tolerogenic phenotype in pDCs that can further educate Tregs and protective antibody responses (**Table 4**).

## CpG-ODN as a Tolerance Inducer in Allergen-Specific Immunotherapy

### CpG-ODN Induces Allergen-Specific Immune Regulation Through DCs

**Figure 2:**
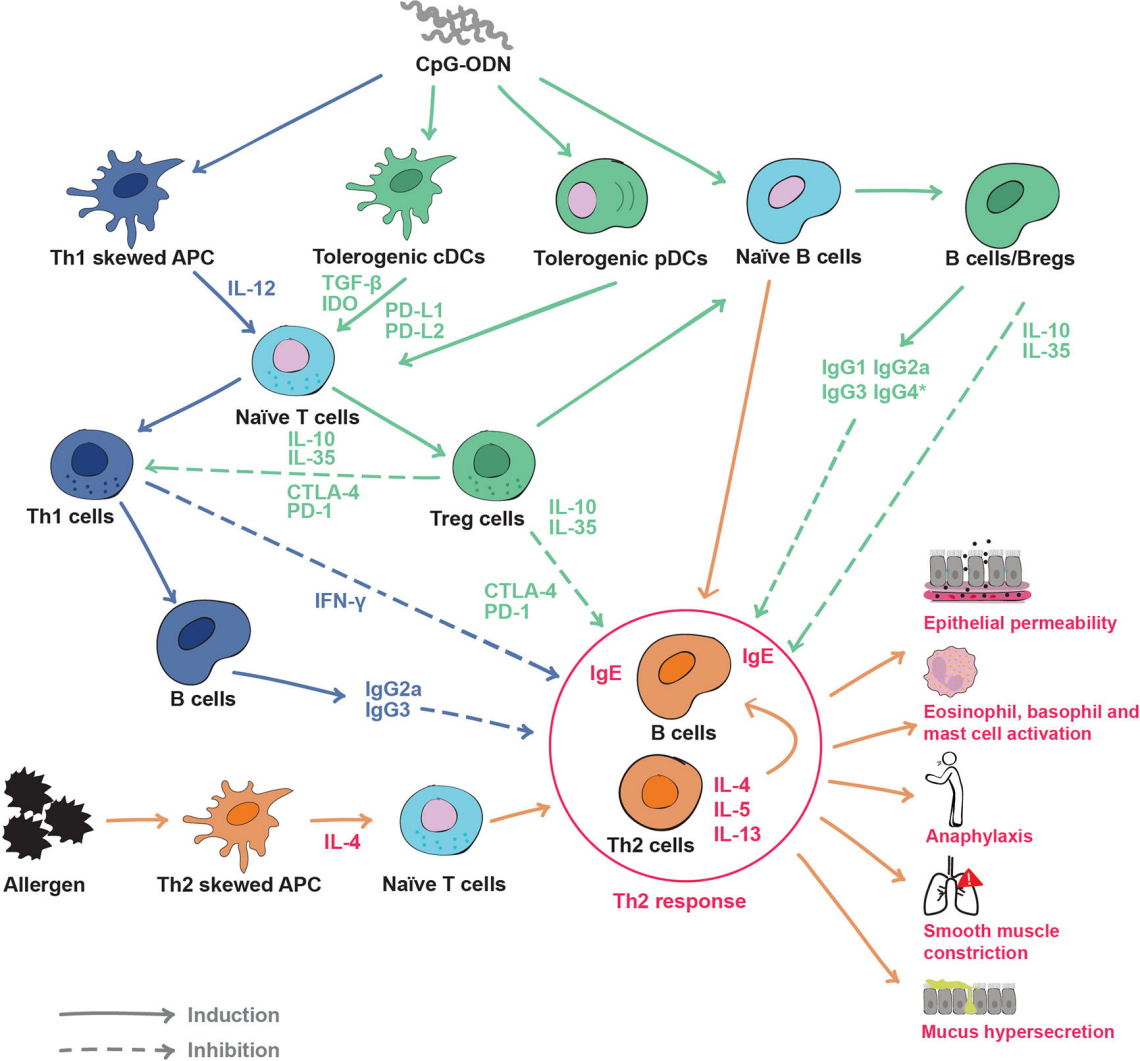
Effects of CpG oligodeoxynucleotides (CpG-ODN) on the immune system and inhibition mechanisms of the Th2 response: In an allergic immune environment (orange and red), allergens skew APCs toward the production of cytokines such as IL-4, prompting naïve T cells into Th2 cells. Th2 cells produce Th2 cytokines (IL-4, IL-5, IL-13) and induce B cells to produce immunoglobulin E (IgE), causing the hallmarks of the allergic disease such as increased epithelial permeability, effector cell activation, anaphylaxis, smooth muscle contraction, mucus hypersecretion etc. CpG-ODN can have different effects on APCs. On one side, it can promote a Th1-like response (blue) by skewing APC to produce IL-12 and then derive naïve T cells to IFN-γ producing Th1 cells. Th1 cells induce B cells to produce neutralizing antibodies such as IgG2a and IgG3. On the other side, CpG-ODN can skew APCs classical dendritic cells (cDCs) and plasmacytoid dendritic cells (pDCs) toward a tolerogenic phenotype (green). Tolerogenic APCs, cDCs and pDCs produce immune regulatory soluble factors such as TGF-β and indoleamine 2,3-dioxygenase (IDO), and express immune modulatory surface molecules like programmed death ligands 1 (PD-L1) and PD-L2. Tolerogenic APCscDCs and pDCs derive naïve T cells to Tregs, initiating an immune regulatory response. CpG-ODN can also engage directly B cells and skew them into a regulatory phenotype (Bregs). Bregs secrete the regulatory cytokines IL-10 and IL-35, as well as neutralizing IgG isotypes. The Th1 response can inhibit Th2 and allergy responses through the production of interferon-γ (IFN-γ) and neutralizing antibodies. However, Treg and Breg cells utilize a wide range of regulatory molecules from soluble IL-10 and IL-35, to the immune checkpoints CTLA-4 and PD-1 to suppress both Th2 and Th1 responses. Solid lines indicate induction, dotted lines indicate inhibition. *IgG4 refers to humans only.

**Figure 3:**
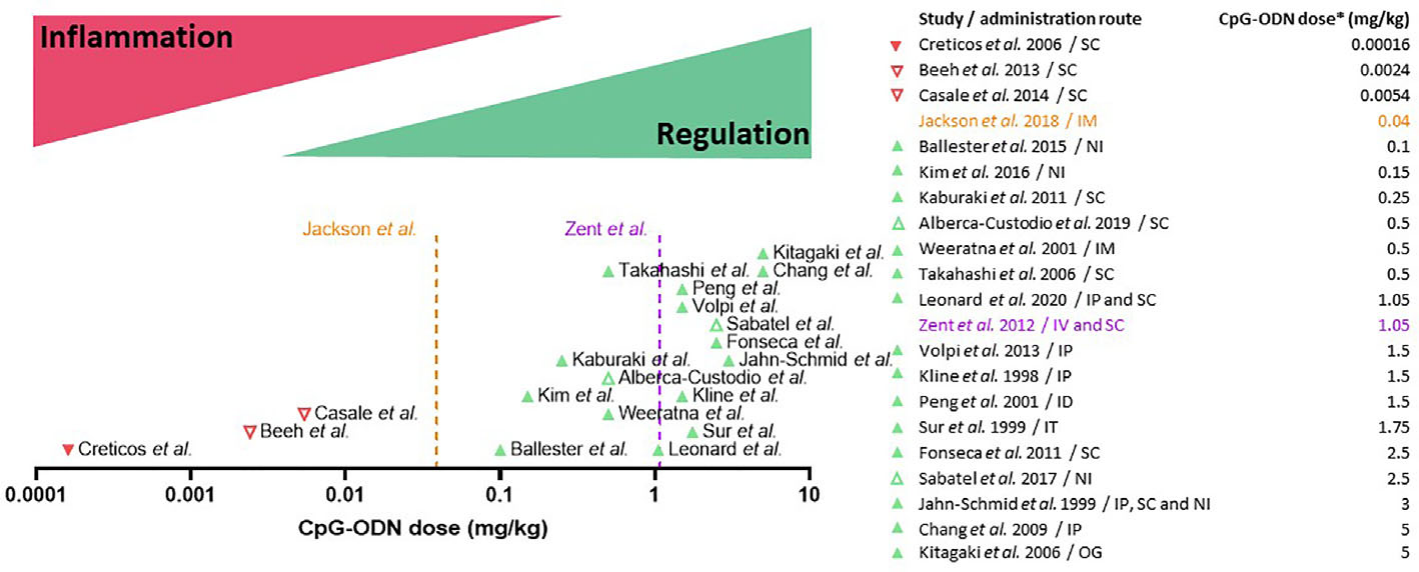
Dual dose effect of CpG oligodeoxynucleotides (CpG-ODN) in AIT: CpG-ODN at low doses triggers immune activation and inflammation, while at high doses it induces immune regulation and tolerance. Clinical (downward-pointing triangles) and pre-clinical studies (upward-pointing triangles) are placed on the X axis (CpG dose in mg/kg body weight, Log10 scale) according to the CpG-ODN dose equivalent used in each study. Unsuccessful or suspended clinical trials and drugs (red triangles) fall under the immune activation/inflammation range of CpG-ODN dosage, whereas most of the successful (green triangles) pre-clinical research studies fall under the regulation/tolerance range. The orange dotted line (Jackson et al., 2018) indicates the dose of B-class CpG-ODN employed to boost the immune response in an FDA-approved vaccine for hepatitis B virus infection. The purple dotted line (Zent et al., 2012) indicates the maximum dose of B-class CpG-ODN that was well-tolerated in humans. Open triangles indicate non-type B CpG-ODN. The table on the right indicates the approximate dose equivalent of CpG-ODN used in each study. *For clinical trials, an average body weight of 75 kg was considered to calculate the CpG-ODN dose. For pre-clinical murine studies, an average body weight of 20 g was considered. IP, Intraperitoneal; NI, Nasal instillation; IT, Intratracheal instillation; ID, intradermal; IM, intramuscular; OG, oral gavage.

As discussed above, DCs and especially pDCs, produce IL-10, TGF-β and IDO upon CpG-ODN stimulation (47, 77), which makes them extremely efficient Treg inducers (**Figure 2**). As innate immunity is crucial to induce a tolerance response of the adaptive immune system, the modulation of APCs such as pDCs and of early-activated adaptive immune cells, such as B cells, is consequently a promising tool for improving AIT. Indeed, Volpi and collaborators (84) showed that CpG-ODN has a dual impact on the immune system. While low CpG-ODN concentrations induced inflammation, high concentrations induced immune regulation and tolerance. Their work suggests the new concept that the same TLR ligand can instruct two distinctive measurable effects on the immune system depending on its concentration/dose (**Figure 1**). Indeed, when CpG-ODN has been used as an adjuvant in vaccination, where a cytotoxic or inflammatory response is needed, the doses used have been low (**Figure 3** and **Table 3**). However, when CpG-ODN has been proven successful in inducing tolerance and treating allergy in animal models, the doses employed have been high (**Figure 3** and **Table 2**). The pivotal immune cells in the research of Volpi et al. were pDCs. The classical role attributed to pDCs in the immune system is the promotion of viral defense through the production of type 1 IFN as a result of TLR danger signal activation by viral genetic material (160). As aforementioned, pDCs have been shown to promote tolerance through several soluble factors and membrane signaling proteins. Although it might seem contradictory at first, several lines of research clearly showed that the stimulation of pDC through CpG-ODN leads to the induction of tolerance (**Figure 2** and **Table 4**). This could be the result of a differential type 1 IFN response triggered by a full-fledged viral infection versus the sole activation of TLR9 through high doses of CpG-ODN. To make a further distinction between pDC-derived type 1 IFN effects from these differential stimulation sources would require additional research. Further evidence in line with their potential for tolerance induction, human pDCs show minimal *NLRP1* and *NLRP3* expression, demonstrating their low capacity for inflammasome activation (175). Accordingly, they can hardly induce adverse inflammatory responses, but are rather programmed to induce tolerance. Indeed, a CD5^+^ CD81^+^ pDC subset was shown to produce no type 1-IFN upon CpG-ODN stimulation, but to induce a strong Treg differentiation (78). Similarly, it has been shown that pDCs primed with CpG-ODN require direct cell contact to induce CD4^+^ CD25^+^ Treg cells (77). Classical dendritic cells (cDCs) have also been shown to promote tolerance through Treg induction in both mice and humans (**Figure 2**). In more detail, a subset of type 2 classical dendritic cells (cDC2a) displays a higher expression of TLR9 and has been shown to induce Treg cell differentiation upon CpG-ODN stimulation (176). Indeed, DCs have been proposed and used to reverse immune-based diseases toward immune homeostasis through tolerance induction (177, 178). Similar to allergy, inflammatory bowel disease (IBD) lacks appropriate immune regulation (179). Studies have shown that CpG-ODN ameliorates IBD hallmarks by inducing tolerance through DCs (180, 181). In accordance with Volpi et al (84)., it was recently shown that AIT using the major cat allergen Fel d 1 together with high doses of CpG-ODN reduces all disease hallmarks in a mouse model of allergic asthma. This was accompanied by an expansion of pDCs and their migration from the injection site to the periphery at early stages of the treatment (79).

### CpG-ODN Induces T and B Regulatory Cells

With regard to immune tolerance induction, CpG-ODN was shown to reduce the Th2 response triggered by aluminum hydroxide when these two adjuvants were co-injected, an effect that was dependent on the activity of MyD88 and IL-10 (182). In agreement with this study, several other studies have pointed out that CpG-ODN is able to induce immune regulatory responses. Already in 1997, bacterial DNA containing unmethylated CG motifs was shown to induce IL-10 production in adherent mouse splenocytes (183). CpG-ODN has also been shown to promote other regulatory cytokines such as TGF-β 3 days after *in vivo* injection in a vertebrate species phylogenetically distant from humans, the fish gilthead seabream (184). TGF-β is crucial for immune regulation and it is one of the three key signals that induce Treg cell differentiation. Furthermore, it is known for its role in tissue repair after inflammation (185). Several research groups have tested different administration routes for CpG-ODN. Kim et al. (80) and Sabatel et al. (76) investigated the nasal installation (NI) route for CpG-ODN application in mice, a proxy for bronchial inhalation in humans. In the first study, the authors showed how pre-treatment with CpG-ODN (medium-high dose: 0.15 mg/kg, **Figure 3**) prevented cockroach-induced allergic asthma in a mouse model (169). The authors found increased levels of IL-10 in lung lysates which was attributed to an increase in the CD4^+^ FoxP3^+^ Treg population. In the second study, Sabatel and colleagues showed as well that NI of CpG-ODN (high dose: 2.5 mg/kg, **Figure 3**) drastically reduced allergic airway inflammation through the secretion of IL-10 (76). In this case, Tregs were not the source of IL-10, but the anti-inflammatory cytokine was secreted by regulatory macrophages recruited after CpG-ODN NI treatment. Both results are not mutually exclusive and could be explained by an initial IL-10 production by regulatory macrophages followed by a successive differentiation of Tregs and production of IL-10. Moreover, after completion of CpG-ODN-based AIT, differentially regulated and de-novo-expressed Tregs were found in the spleen of CpG/AIT-treated mice (79), including Treg subtypes which are known to specifically suppress Th2 responses (186, 187). In addition to Tregs, Bregs are also of crucial importance in the success of AIT (40). As B cells express higher levels of TLR9 compared to other immune cells, they are strongly activated by CpG-ODN. Adoptive transfer of splenocytes from mice treated with CpG-ODN conferred resistance to allergic inflammation in a mouse model (188). The authors found that the reduction of the allergic response was dependent on IL-10 producing follicular B cells (B220^+^ CD19^+^ CD23^+^ IgM^+^ CD40^+^ MHCII^hi^), indicating that CpG-ODN-stimulated B cells have a role in promoting immune tolerance in the context of allergy. Furthermore, it has been shown by Ticha et al. that IL-10 producing B cells induced by CpG-ODN have a distinct expression of tumor necrosis factor receptor 2 (TNFR2) (82), which could also be confirmed in the murine CpG-ODN-based AIT model where B cells expressed higher levels of TNFR2 at the injection site (79).

### CpG-ODN Induces Protective Immunoglobulins and Improves Epithelial Integrity

As explained above, one of the important mechanisms of AIT in reducing allergic symptoms is the production of protective immunoglobulins by B cells such as IgGs and IgA (46). Indeed, B cell stimulation with CpG-ODN induces the production of neutralizing immunoglobulins such as IgG2a, IgG2b, IgG3 and IgA in mice (87–89, 102). Furthermore, CpG-ODN inhibits IgE production *in vivo* (89, 90). CpG-ODN has also been shown to enhance protection from allergic diseases through co-activation of non-immune cells. For instance, CpG-ODN improves tight junctions in airway epithelial cells, a cell type playing a crucial role in the homeostasis of lungs and the prevention of airway sensitization by allergens (189–191).

### CpG-ODN Induces Th1 Responses

As introduced in the previous section, CpG-ODN has been used as an adjuvant in AIT formulations to target the innate immune system and therefore increase therapeutic effects of AIT. Among others, CpG-ODN has been described to induce a Th1 immune response. Indeed, the first assays where CpG-ODN was used *in vitro* reported production of IFN-γ and IL-12, the canonical cytokines produced in Th1 environments (170, 173, 192). Interestingly, *in vivo* preventive allergy treatment with CpG-ODN has been shown to induce an immutable IFN-γ-dependent Th1 response, which prevented the establishment of a subsequent Th2 allergic response (98). Comparably, when mice are primed with the hepatitis B major surface antigen (HBsAg) using aluminum hydroxide as adjuvant, a Th2 response is induced, but when the same mice are subsequently treated with CpG-ODN and HBsAg, the CpG-ODN boost is able to induce a Th1 response, overwriting the pre-established Th2 response (88). Accordingly, the induction of Th1 responses has been proposed in the past as a possible cellular immune mechanism to treat allergic diseases (193, 194).

### CpG-ODN-Based Allergen Specific Immunotherapy Considerations

As depicted above, different routes of administration of CpG-ODN such as intraperitoneal (IP), subcutaneous (SC), epicutaneous (EPI) or NI have been applied to ameliorate allergic disease phenotypes in murine models and humans (**Tables 2** and **3**). Because of this disparity in the approaches with varying outcomes, additional studies are needed to find the optimal route of administration to be used in combination with the most effective dose of CpG-ODN and the most advantageous AIT treatment regimen. In common clinical practice, the subcutaneous route of injection is preferred. However, non-invasive delivery routes are not to be neglected, since AIT has been proven to be successful using a sublingual approach *via* sublingual immunotherapy (SLIT) (195). SLIT has been shown to engage potential tolerance inducing immune cells such as DCs and pDCs or Tregs in the oral mucosa (196, 197). Hence, the usage of a CpG-ODN-based AIT *via* other administration routes such as SLIT would increase its therapeutic value. Although both, Th1 and Tregs are known to suppress allergy (186) (**Figure 2**), it is undoubtedly preferable to induce a Treg/Breg reaction than a Th1 response since Th1 cells have been associated with undesirable side effects such as inflammation or even with autoimmunity (198). Because of these considerations, immune tolerance induction should be preferred to treat allergic diseases over induction of Th1 cells to replace the existing Th2 response (**Figure 2**). Some of the CpG-ODN-based treatment strategies presented in this review use CpG-ODN as an immunomodulator in an allergen-free formulation. This creates an unspecific tolerance induction which is usually not long-lasting due to the lack of immune memory. On the contrary, when CpG-ODN is used together with the allergen, the resulting tolerance induction is allergen-specific and long-lasting, as it is supported by immune memory (31, 40). Accordingly, although treatments for allergic diseases using only CpG-ODN seem to confer a certain level of short-term immune tolerance (76, 104, 105) (**Table 2**), CpG-ODN-based AIT formulations should be preferred in order to induce specific and durable tolerance to the allergen(s). An overview of the literature would also be strongly in favor of using CpG-ODN with allergen as a prophylactic vaccination-like treatment for allergic diseases by inducing either a Th1 or a Treg response (199), thus preventing the subsequent development of a pro-allergic Th2 environment. However, this does not reflect the situation in the clinic, where allergic patients, who are already sensitized and present with a pre-established symptomatic disease, consult the clinicians. Therefore, a prophylactic treatment for allergic diseases represents a future scenario for those in the general population, who are at high risk of developing allergic disease. Based on all current data, CpG-ODN-based therapies for allergy have the potential to be successful as active treatment in AIT when used in a novel formulation. Therefore, this should be the immediate next step to follow when designing novel therapeutic strategies for allergic diseases using CpG-ODN. Interestingly, it has been shown that when pDCs and B cells are stimulated with CpG-ODN, their TLR9 expression is downregulated within 12 h (167), probably due to negative feedback. Consequently, repeated CpG-ODN injections in a short period of time would trigger a much lower effect. Similarly, key cells such as B cells, DCs and macrophages change their TLR9 expression according to the circadian rhythm in mice, showing a higher expression coinciding with the mouse active phase (200). According to this knowledge, a timely spaced injection schedule during the active human phase would be preferable to maximize TLR9 signaling in critical immune cells, thus enabling the desired effect of the adjuvant.

The analysis of the literature discussed above, which is graphically summarized in **Figures 2** and **3**, encouraged us to propose that CpG-ODN used in the adequate conditions and concentrations might be a strong inducer of a tolerogenic immune response for allergy and other inflammatory disorders, acting by modulation of various layers of the immune system (**Figure 2**). Other licensed adjuvants such as Al(OH)_3_ or MPL have been used and tested in the context of AIT. However, they induce pro-inflammatory responses such as inflammasome activation and Th1 responses (**Table 1**) that can revert the underlying Th2 immune phenotype but lack the tolerance promoting properties of CpG-ODN.

## CpG-ODN as an Adjuvant for Allergen Specific Immunotherapy

### Reconsidering CpG-ODN for Allergen-Specific Immunotherapy

According to the studies discussed in the previous sections, CpG-ODN used in the appropriate conditions induces tolerance in the immune system through various cells and molecular mechanisms. With this systematic review, we intend to support the reconsideration of CpG-ODN as an adjuvant for AIT formulations with the purpose to enhance its therapeutic effects and overcome the aforementioned challenges of AIT such as side effects and burdensome treatment schedules. Indeed, CpG-ODN has shown potential to alleviate the burden of allergic diseases through various immune processes. Multiple lines of evidence have shown that CpG-ODN exerts its immune modulating effects mainly through two complementary mechanisms in many pre-clinical studies (**Table 2**). On the one hand, the induction of an immune regulatory response in the form of tolerogenic pDCs, Breg and Treg cells. On the other hand, the generation of allergen-specific neutralizing antibodies that block allergen binding by specific IgE (sIgE) and thus inhibit allergy effector cells such as eosinophils, basophils or mast cells (**Figure 2**). These immune-modulatory properties of CpG-ODN would ultimately promote immunological and clinical tolerance to the allergen.

### TLR-Ligand Interference as a Consideration in the Design of CpG-ODN-Based Allergen-Specific Immunotherapy

A crucial aspect in the design of CpG-ODN-based AIT formulations is the possible interference with other immunostimulatory PAMPs, especially with other TLR ligands such as LPS. Indeed, the immune system is a complex network of receptor-ligand interactions and connected intracellular signaling pathways. Many TLR downstream signaling molecules, particularly kinases, are shared between the TLR family members (125, 143). Consequently, a secondary TLR-stimulation, in parallel to TLR9 activation by CpG-ODN, could alter the outcome and quality of TLR9 signaling. In the case of CpG-ODN employment as an adjuvant for AIT, the desired result is the induction of immune tolerance without inducing an inflammatory response. The presence of residual LPS would activate the TLR4 signaling pathway, which competes with concurrent TLR9 signaling for downstream signaling molecules, thus biasing and deviating the tolerogenic pathway induced by CpG-ODN. Indeed, it has been shown that co-activation of TLR9 and TLR4 induces a strong inflammatory signal in form of TNF-α and IL-12 secretion (201) (**Figure 1**). Among other reasons, such an interference by low amounts of residual LPS (**Figure 1**) in the natural ragweed allergen Amb a 1 could explain why previous AIT clinical trials using CpG-ODN were unsuccessful (26, 111). However, with the appropriate knowledge from preclinical studies, one has the ability to influence and activate only specific immune cells and pathways to achieve the desired immunotherapeutic precision yield.

### High Doses of CpG-ODN Induce Immune Regulation

Another essential factor in CpG-ODN formulations is the dose. As outlined before, to achieve tolerance induction, high CpG-ODN concentrations and doses are needed (84). Indeed, a principal pattern can be extracted when systematically analyzing the numerous clinical trials (**Table 3**) and pre-clinical studies (**Table 2**) in which CpG-ODN has been used in the context of allergic diseases (**Figure 3**). On the clinical side, the maximum CpG-ODN dose used for allergy treatment in humans equals to approximately 0.0054 mg/kg per injection, repeated over 7 injections (113). However, this clinical trial showed no benefit in patients, and was prematurely terminated with no further follow-up. Other clinical trials in humans used even lower dose equivalents of CpG-ODN adjuvant (111, 112). In contrast to human studies in the allergy field, CpG-ODN doses used in preclinical allergy studies were markedly higher and more successful. Indeed, many of the animal research studies with positive findings used at least a 10-fold higher CpG-ODN dose per injection compared to the less successful clinical trials (**Table 3** and **Figure 3**). The fact that lower CpG-ODN doses were employed in human clinical trials could explain the lack of CpG-ODN activity as AIT adjuvant. The only marketed pharmaceutical product currently using CpG-ODN is a subunit vaccine for HBV that has recently received approval by the FDA (120). Vaccines necessitate firm and sustained immune activation, sometimes also referred to as inflammation, to induce protection against pathogens. Interestingly, the new HBV vaccine utilizes a low dose of CpG-ODN to induce an enhanced immune response against HBV. However, this relatively low dose of 0.04 mg/kg CpG-ODN per injection, which was shown by Jackson et al. (120) to display superior vaccination adjuvant activity over aluminum hydroxide for HBV prevention, is still higher than the CpG-ODN dose equivalents previously applied in clinical trials to treat allergy (**Figure 3**). In contrast, CpG-ODN has been successful in reducing allergy burden in animal models when used at higher doses (**Table 2**). Based on the analysis presented (**Figure 3**), we suggest to reconsider CpG-ODN as an AIT adjuvant, but at higher doses than previously applied in humans, to treat Th2-/IgE-mediated allergic diseases. Intriguingly, the maximum dose of well-tolerated B-type CpG-ODN after intravenous injections in humans as shown by Zent et al. is in the range of tolerance-inducing CpG concentrations reported by others (79, 84, 114) (**Figure 3**).

### CpG-ODN to Treat Other Non–IgE-Mediated Immune Diseases

Furthermore, one could think of applying the immune regulatory properties of CpG-ODN not only in allergy therapy, but also to treat other diseases in which the immune system is dysregulated such as multiple sclerosis (MS), where the induction of antigen-specific immune tolerance would be needed. As similar or even identical immune tolerance checkpoint mechanisms have been identified across immune-mediated diseases (202, 203), the co-administration of the self-antigen myelin oligodendrocyte glycoprotein (MOG) together with high doses of CpG-ODN could potentially reduce the autoimmune reaction observed in the experimental autoimmune encephalomyelitis (EAE) mouse model, setting the basis for an antigen-specific tolerance induction therapy in MS and other autoimmune diseases.

### Route of Administration for CpG-ODN-Based AIT

As aforementioned, CpG-ODN-based AIT has been applied to animal disease models using a wide variety of routes of administration, such as IP, NI, subcutaneous (SC), intradermal (ID), intramuscular (IM) and oral gavage (OG) routes (**Table 2**). While the dosage appears to be key in order to achieve immune tolerance in the context of CpG-ODN-based AIT, the role of the administration procedure is less clear. The application of the therapy *via* a particular method of delivery to treat a tissue-specific allergy phenotypes, for example intranasal or intrabronchial administration to treat airborne allergies, is an interesting point to consider. Indeed, some animal studies successfully treat airway allergy using intranasal application of CpG-ODN-based AIT (76, 80, 108) (**Table 2**). However, a comprehensive analysis of the pre-clinical studies (**Table 2**) suggests that the route of administration does not always have to target the tissue or organ affected by the allergy in order to achieve its therapeutic effect. Indeed, many of the animal studies described in **Table 2** treat respiratory allergies through an IP, SC or ID administration (79, 103–106). Furthermore, in clinical practice the SC route has proven its efficacy to treat airway allergies, indicating that the administration method can be independent of the allergy phenotype to be cured. Nevertheless, novel delivery methods such as sublingual or intrabronchial therapies can result in improved efficacy and ease of application in clinical studies (23, 26, 91, 96, 196), suggesting that an optimized route of application could also improve CpG-ODN-based AIT. However, since the dose of CpG-ODN is the primary determinant for treatment efficacy, studies would need to be fine-tuned regarding the dosage for each novel route of administration without compromising safety.

## CpG-ODN – Safety Considerations and Other Applications

### CpG-ODN as an Adjuvant for Pathogen and Cancer Vaccination

Besides its role as immunomodulatory agent in the treatment of asthma or allergic rhinitis and as adjuvant in AIT, CpG-ODN has been applied in other sectors of pharmaceutical research, mainly as adjuvant for vaccines against pathogens, and as immune-enhancing drug for cancer treatment (204). CpG-ODN has been used to confer protection through vaccination against a wide variety of pathogens (205), including bacteria (199). Indeed, CpG-ODN can induce pathogen-specific IgM enhancing phagocytic activity against *S. aureus* (206). Furthermore, it has been tested with success in a phase I clinical trial of a BCG multi-subunit vaccine against *M. tuberculosis* (121) (**Table 3**), where relatively low CpG-ODN dose equivalents of 0.001 mg/kg (75 μg per injection) and 0.02 mg/kg (150 μg per injection) induced robust IFN-γ and IgG responses. As mentioned above, CpG-ODN can activate and enhance NK cell function, a characteristic that some authors have associated with enhanced anti-viral properties. For instance, CpG-ODN has been shown to effectively fight alphavirus encephalitis in neonatal mice (162). Moreover, CpG-ODN has also been shown to be efficacious as an adjuvant in vaccines against viruses, for example, by generating a humoral protective response to the HBV surface antigen HBsAg (88), which subsequently lead to the development of an FDA-approved vaccine against HBV that was superior compared to an established aluminum hydroxide-based HBV vaccine (120). Similarly, pre-clinical models of vaccination against viruses have also used CpG-ODN as adjuvant in their formulations, such as in vaccination studies targeting the influenza virus strain H1N1 (140). With regard to cancer treatment, CpG-ODN has been extensively used for its immune enhancing properties (136). The first reported case where bacteria were used against cancer was published in 1893 by Dr. William Coley using live bacteria injected directly into tumor tissue (207). Later, Dr. Coley utilized heat-killed bacteria with similar effects (208). These observations sparked the research on bacterial compounds such as CpG-ODN for cancer treatment (209). It has been shown that CpG-ODN potentiates both innate and adaptive anti-tumor immunity, mainly by enhancing NK cell and CD8 T cell cytotoxic activity (81, 210). Among other successful applications in the cancer field (**Table 3**), CpG-ODN was used as prophylactic treatment for reducing brain metastasis through microglia activation (211), or in combination with M362, a TLR6 ligand, with which it potentiates CD8 T cell response against breast cancer cells (212).

### Safety Considerations Regarding CpG-ODN

Although CpG-ODN showed a good safety profile, there have been some concerns regarding the potential triggering of autoimmune diseases by this TLR9 agonist (136, 213). For instance, CpG-ODN has been associated with the induction of arthritis *in vivo* when injected into animal joints (214, 215). Similarly, when bacterial DNA containing CG motifs is injected interstitially into the meninges of rats, signs of meningeal inflammation appear on the histopathologic level (216). However, the injection of CpG-ODN directly into articulations or meninges is by no means foreseen in a CpG-ODN-based AIT. Furthermore, neither of these effects have been observed in clinical trials using CpG-ODN (**Table 3**) nor in the individuals that received the approved CpG-ODN-adjuvanted HBV vaccine. Another possible safety concern is that CpG-ODN could activate autoreactive B cells to produce double stranded DNA (dsDNA) autoantibodies and cause autoimmune diseases similar to systemic lupus erythematosus (SLE). However, only very few cases of anti-dsDNA antibodies or other signs of autoimmune disease have been reported in subjects receiving CpG-ODN (136) with no clear link to the adjuvant administration. Similarly, and although CpG-ODN was suspected to exacerbate and maintain dextran sulfate-induced colitis in a mouse model of IBD (217, 218), a later publication found CpG-ODN to be protective for IBD (180).

### Safety Considerations Regarding High Dose CpG-ODN

Like many other drugs and compounds, CpG-ODN used in very high doses (>2.4mg/kg) and repetitive injection schemes (daily injections) showed toxicity in mice (219). However, in lower and more conventional dosages, CpG-ODN has been recognized to be a safe compound in humans with relatively benign effects in terms of toxicity (**Table 3**). Consequently, it has been recently approved by the FDA as adjuvant for a HBV vaccine (120). Some of the CpG-ODN-derived side effects described in clinical trials (**Table 3**) are probably caused by its rapid systemic distribution upon injection. Furthermore, all the clinical trials using CpG-ODN for allergy treatment rely on subcutaneous administration (**Table 3**), a route known for its systemic distribution of the compounds through capillaries and lymphatic vessels (220). This presents a challenge regarding possible mild, but tolerable side-effects, which needs to be addressed, since the tolerogenic effects of CpG-ODN are only observed at relatively high doses (**Table 2**, **Figure 3**). However, a high concentration of CpG-ODN does not necessarily have to be systemic and can also act locally to promote immune tolerance. According to *in vitro* experiments (84, 132), a local but high concentration of CpG-ODN should have the same effect on key immune cells such as pDCs and B cells, without causing systemic toxicity. Several pharmacological strategies can be employed to achieve a high local concentration, thus preventing a rapid systemic distribution of compounds. One solution is the usage of drug delivery matrices forming a depot and assuring a slow substance release, like hydrogels, nanoparticles or liposomes (221, 222). When injected subcutaneously, these substances form a depot that encloses all the compounds of the AIT formulation. In case of a CpG-ODN-based AIT, such an approach would allow for a high local concentration of CpG-ODN, avoiding its rapid systemic release, thereby minimizing side effects, while allergens are also progressively released. Moreover, these drug delivery systems are composed of biocompatible and biodegradable compounds, which makes them safe and suitable as part of pharmaceutical products (223, 224).

Altogether, these data indicate that CpG-ODN can be used as an adjuvant in a variety of therapeutic strategies. Based on current knowledge, CpG-ODN-based AIT using high doses of CpG-ODN to induce tolerance would be a safe and potentially beneficial therapy for patients suffering from moderate or severe allergic diseases.

## Conclusion

AIT has been used for almost 110 years and remains to this date the only disease-modifying treatment for allergic diseases that can provide allergy cure. It presents several advantages over symptomatic therapies such as its cost-effectiveness and long term tolerogenic effects. Despite being a successful therapy in many aspects, AIT still has unmet needs to be faced and solved. One of the most affordable, suitable, practical and promising strategies relies on the use of optimized adjuvants to boost the therapeutic effects of AIT. CpG-ODN has been used in the past as an adjuvant for AIT with limited success that could be explained by the use of rather low doses of CpG-ODN at that time, probably due to caution and safety concerns when applying it in humans. Based on newer evidence, CpG-ODN induces tolerance in high doses. Therefore, we argue for its reevaluation as a potentially beneficial adjuvant in AIT. We propose that doses between 0.5 and 1.5mg/kg, dependent on the administration route, should induce the desired immune tolerance toward the allergen with a minimal risk of adverse effects. Specifically, B-class CpG-ODN is capable to induce tolerance with very low unwanted reactogenicity, making it a strong candidate for further translation into the clinic. Indeed, by combining CpG-AIT and novel drug delivery systems, even higher doses of CpG-ODN could be tailored to be well tolerated, thus aiding to further improve the safety of CpG-ODN-based AIT. The route of administration is another important factor to be considered. Pairing the route of administration with the organ-specific allergy phenotype could be a promising strategy to enhance the tolerance-promoting capacity of CpG-AIT. Further research will help to unveil the regulatory immune mechanisms of CpG-ODN-based AIT and design better strategies to specifically target key immune cells such as pDCs and other APCs. Overall, CpG-ODN used in AIT has the potential to greatly benefit allergic patients as it represents a safe, effective and possibly curative approach for allergic diseases.

## Author Contributions

All authors contributed to the conception and writing of the manuscript as well as read and approved the final manuscript. All authors contributed to the article and approved the submitted version.

## Funding

This work was supported by the Ministry of Higher Education and Research of Luxembourg through the intramural research program of the Luxembourg Institute of Health. GM and MO were supported by the Luxembourg National Research Fund (FNR) through the FNR-PRIDE program NEXTIMMUNE for doctoral education (PRIDE/11012546/NEXTIMMUNE). AP was supported by the Lions Club Luxembourg, through the Action Lions Vaincre le Cancer association.

## Conflict of Interest

MO reports personal fees from Allergy Therapeutics/Bencard, Great Britain/Germany; Thermo Fisher Scientific, Sweden; Siemens Healthcare Diagnostics, Germany; Hitachi Chemical Diagnostics, USA; and Hycor Diagnostics, USA outside the submitted work; and is Scientific co-founder of the academic biotech spin-offs PLS-Design GmbH, Hamburg, Germany and Tolerogenics SARL, Luxembourg. CL and MO report to be co-inventors on two patents pending on hydrogel-embedded oligodeoxynucleotides as tolerogenic adjuvant for subcutaneous immunotherapy and induction of allergen-specific Tregs prior to oral or sublingual immunotherapy of food allergy. LK reports receiving research grants from Allergy Therapeutics/Bencard, Great Britain/Germany; ALK-Abelló, Denmark; Allergopharma, Germany; ASIT Biotech, Belgium; AstraZeneca, Sweden, Biomay, Austria, Boehringer Ingelheim, Germany, Circassia, USA; Stallergenes, France; Cytos, Switzerland; Curalogic, Denmark; HAL, Netherlands; Lofarma, Italy; Mylan, USA; Novartis, Switzerland, Leti, Spain; ROXALL, Germany; GlaxoSmithKline (GSK), Great Britain; Sanofi, France and/or has served on the speaker’s bureau or was consulting for the above mentioned pharmaceutical companies. LK is the current President of AeDA (German Society of Applied Allergology), Vice-President of the German Academy for Allergy and Environmental Diseases and Chair of EAACI ENT section.

The remaining authors declare that the research was conducted in the absence of any commercial or financial relationships that could be construed as a potential conflict of interest.

## Abbreviation

AIT, allergen-specific immunotherapy; Al(OH)_3_, aluminum hydroxide; AP1, activator protein 1; APC, antigen presenting cell; Breg, B regulatory cell; cDC2a, type 2a classical dendritic cell; cDCs, classical dendritic cell; CpG-ODN, CpG oligodeoxynucleotide; CTLA4, cytotoxic T-lymphocyte-associated protein 4; DC, dendritic cells; dsDNA, double stranded DNA; EPI, epicutaneous; FoxP3, forkhead box P3; Gata3, GATA Binding Protein 3; GM-CSF, granulocyte-macrophage colony-stimulating factor; HBsAg, hepatitis B major surface antigen; IBD, intestinal bowel disease; ICOS-L, inducible costimulatory ligand; ID, intradermal; IDO, indoleamine 2,3-dioxygenase; IFN, interferon; Ig, immunoglobulin; IKK, IκB kinases; IL, interleukin; IM, intramuscular; IP, intraperitoneal; IRAKs, IL-1R-ssociated kinase; IRF, interferon regulatory factor; LAG3, lymphocyte-activation gene 3; LPS, lipopolysaccharide; MAPK, mitogen-activated protein kinase; mDC, myeloid dendritic cell; MLP, monophosphoryl lipid A; MyD88, myeloid differentiation factor 88; NF-κB, nuclear factor kappa-light-chain-enhancer of activated B cells; NI, nasal instillation; OG, oral gavage; PAMP, pathogen associated molecules pattern; pDC, plasmacytoid dendritic cell; PD-L1, programmed death ligand 1; PD-L2, programmed death ligand 2; PLGA, poly-(lactic-co-glycolic acid); PRR, pattern recognition receptor; SC, subcutaneous; SLE, systemic lupus erythematosus; SLIT, sublingual immunotherapy; TGF-β, transforming growth factor beta; Th1 cells, T helper cell type 1; Th2 cells, T helper cell type 2; TIR, toll/IL-1 receptor domain; TLR, toll-like receptor; TNFR2, tumor necrosis factor receptor 2; TNF-α, tumor necrosis factor alpha; TRAF, tumor necrosis factor receptor-associated factor 6; Treg, T regulatory cell; TRIF, TIR-domain-containing adapter-inducing interferon-β.
